# A Framework for Describing the Influence of Service Organisation and Delivery on Participation in Fetal Anomaly Screening in England

**DOI:** 10.1155/2017/4975091

**Published:** 2017-03-22

**Authors:** Hyacinth O. Ukuhor, Janet Hirst, S. José Closs, William J. Montelpare

**Affiliations:** ^1^Department of Public Health, Saudi Electronic University, P.O. Box 93499, Riyadh 11673, Saudi Arabia; ^2^School of Healthcare, University of Leeds, Baines Wing, Room 3.10, Leeds LS2 9JT, UK; ^3^School of Healthcare, University of Leeds, 2.25 Baines Wing, Leeds LS2 9UT, UK; ^4^Margaret and Wallace McCain Chair in Human Development and Health, University of Prince Edward Island, Charlottetown, PE, Canada C1A 4P3

## Abstract

*Objective*. The aim of this research was to explore the influence of service organisation and delivery on providers and users' interactions and decision-making in the context of Down's syndrome screening.* Methods*. A qualitative descriptive study involving online interviews conducted with a purposive sample of 34 community midwives, 35 pregnant women, and 15 partners from two maternity services in different health districts in England. Data were analysed using a combination of grounded theory principles and content analysis and a framework was developed.* Results*. The main emerging concepts were organisational constraints, power, routinisation, and tensions. Providers were concerned about being time-limited that encouraged routine, minimal information-giving and lacked skills to check users' understanding. Users reported their participation was influenced by providers' attitudes, the ambience of the environment, asymmetric power relations, and the offer and perception of screening as a routine test. Discordance between the national programme's policy of nondirective informed choice and providers' actions of recommending and arranging screening appointments was unexpected. Additionally, providers and users differing perceptions of emotional effects of information, beliefs, and expectations created tensions within them, between them, and in the antenatal environment.* Conclusions*. A move towards a social model of care may be beneficial to empower service users and create less tension for providers and users.

## 1. Introduction

The National Health Service (NHS) antenatal Down's syndrome screening programme (DSS) in England and Wales is publicly funded and is governed by the UK National Screening Committee (UK NSC) as part of the Fetal Anomaly Screening Programme [1]. For over a decade, it has been a national policy to offer universal Down's syndrome screening to all pregnant women and their partners when present [2], guided by a nondirective informed choice policy. The main reason for the policy is to protect providers from the notion of eugenics, as it may involve users deciding whether to terminate or keep an affected pregnancy. DSS is offered by frontline providers, usually community midwives with other routine antenatal tests at the first antenatal appointment (booking visit). The current DSS screening programme consists of the combined screening test which comprises a blood test and an ultrasound scan to measure the nuchal translucency. This determines a woman's risk for having a baby with Down's syndrome and is undertaken before 12 weeks. The quadruple test (serum test) is offered to women in the second trimester who present late for antenatal care (after 12 weeks).

The policy regarding Down's syndrome screening advocates that participation should be underpinned by “informed choice” [1]. This entails the provision of high quality, complete, up-to-date information about screening and ensuring users decision-making processes are autonomous and free from external influences. However, there is evidence [3] that even when patients are well educated and well informed about their treatment options, many still find it difficult to engage meaningfully in decision-making about their care. The literature broadly suggests that service providers are supportive of ensuring pregnant women have choices about all aspects of health care, in particular, antenatal screening, but there is inconsistency in such operationalisation [4–10]. Additionally, a UK government White Paper [11] on the NHS expressed concerns that the organisational structure of the NHS is excessively bureaucratic and exerts top-down control. Users are expected to fit around services, rather than services around them. These concerns have prompted a debate among politicians about the influence of NHS organisational structures and processes on users' decision-making, but this issue has not been comprehensively investigated in Down's syndrome screening. Existing studies have focused on the sociodemographic differences among women to account for their participation in the programme, with inconclusive evidence [6, 12–18].

Research conducted in the UK [9] reported that midwives experienced challenges such as time constraints and often resorted to the use of photographs of children with Down's syndrome. Another challenge encountered was when women asked for advice. Further, the literature [7] suggests that some parents were given too much information which did not help with decision-making, having gaps in information, and providers experienced challenges in providing information to parents. The potential ramification of these findings is that the operationalisation of the first trimester DS screening programme may be proved challenging due to some service organisational issues. Additionally, prior research [19] suggests health professionals' opinions and attitudes influenced women to accept the screening test. This was not surprising as the uptake rate of first trimester screening was 95% at the study site. What was surprising was that the majority of the women reported they made the decisions based on their moral values and beliefs as previously noted in Williams et al. [20]. However, Paul (1998) cited in Seavilleklein [21], Dormandy et al. [22], Dormandy et al. [23], and Skirton and Barr [24] suggest that pregnant women may make decisions that are at variance with their attitudes, beliefs, and values owing to the influence of health professionals. Similarly, other studies found the personal opinions and attitudes of health professionals influenced women's decision-making about screening [4–6, 19, 25–31], but research conducted in the UK and Netherlands found no influence of midwives' attitudes on women's choices [30, 32, 33]. These prior studies clearly indicate there is scope for further research into service organisational influences on users' decision-making processes. Also, many of these studies predate the introduction of universal screening in the UK [4, 5, 25, 34, 35] whilst others reported on second trimester screening for Down's syndrome [6, 10]. Other available literatures on first trimester screening were conducted with General Practitioners (GPs) in Australia [36], Canada [37], and Hong Kong [38]. The findings may not necessarily be relevant to the NHS service within England where community midwives are the main providers.

Moreover, service organisational influence on participation in screening is becoming more important to understand, as there is marked variation in participation rates in DS screening within and between countries with similar demography and screening policies [39–41]. Variation in uptake rates of between 22.7% and 73.9% has been reported in the North of England [42]. It is also anticipated that Noninvasive Prenatal Testing (NIPT) is likely to become available in the NHS and uptake rates are expected to increase by virtue of the screening test having better predictive outcomes [43, 44].

Overall, there is a consensus that some organisational issues have an influence on women participation in screening. Yet studies that have comprehensively explored broader service organisational and delivery influences on decision-making processes in the DS screening context based on pregnant women, their partners, and midwives perspectives are notably lacking in the literature. Thus, we need to understand organisational influences on users' participation to inform and support policy and practice as a means to improve service delivery in the NHS. This study aimed to gain deeper understanding of the influence of service organisation and delivery on users' decision-making processes using the Donabedian healthcare delivery model.

## 2. Methods

**Figure 1:**
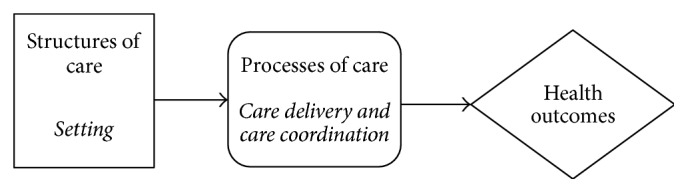
The Donabedian healthcare organisation and delivery model [51].

The Internet is increasingly being used in maternity services to explore the perceptions and experiences of service users and providers [45, 46]. A qualitative descriptive study involving online interviews was employed adopting the Donabedian model as a framework (Figure 1). The Donabedian healthcare organisation and delivery model [47, 48] is one of the most frequently used models to evaluate the quality of healthcare services including nursing and midwifery services [49, 50]. The model assumes a linear relationship between structures, process, and outcome variables. Structures denote the attributes of the settings in which service or care is provided. This includes the physical setting, such as the facilities and equipment available, and the care environment, such as the ambience of the consulting rooms. It also includes the healthcare professional's attributes, such as professional competence, interpersonal skills, commitment to the role, and personal characteristics. Process refers to activities involved in giving and receiving care. It includes a range of healthcare activities that make up caring as perceived by patients such as providing information, reassurance, showing concern, and respect. The model was adopted to explore whether organisational issues that are associated with provision and utilisation of hospital resources were relevant in the DS screening programme using online data collection. This enabled the generation of a framework of organisational factors that affect decision-making processes in the antenatal context.

### 2.1. Sample and Data Collections

A purposive sample of two maternity services was identified from routine reporting of regional data in England [42]. Two NHS Trusts reflected high uptake (city maternity service) and low uptake (district maternity service) of the antenatal Down's syndrome screening. In both locations first trimester combined screening test was the preferred screening test.

Recruitment and data collection took place between March 2012 and March 2013. A purposive sampling method was used to recruit women and their partners who have been offered screening, by the lead author. Ultrasonographers introduced the survey to potential participants (pregnant women and their partners) at the fetal assessment appointment; those interested in receiving more information met the researcher at the hospital after the fetal anomaly scan. At this point interested potential participants were offered a hard copy of the information sheet and the opportunity to ask the researcher questions. Those who agreed to participate were invited to access the online study including the completion of a consent agreement. The information sheet was also provided online. Women were required to be aged 16 years or over, as the care pathway for women below 16 years of age was different; that is, antenatal appointments were generally longer by a specialist midwife to enable tailor-made information-giving. In addition, potential participants must have been offered DS screening in either setting. All community midwives in both settings were invited to participate via letters distributed by local team leaders. Participants who accessed the online survey and decided to withdraw could exit the study at any time by clicking on an “exit” button. Electronic reminders were not sent to such participants.

Data collection was by way of asynchronous online survey using open questions in an “interview” style. In asynchronous online interview, an individual participant may choose to respond to the questions at any convenient time. A rationale for using online interview was to be less intrusive than traditional interviews and provide flexibility and control for all participants. The literature suggests that some women find it difficult to criticise health professionals in face-to-face interviews [52–55]; hence, this method was adopted to gather anonymous comments which could be either positively or negatively framed.

Pregnant women and their partners accessed the online interview which included vignettes, open-ended questions, and written and photographic prompts which set the context, encouraged reflection, and provide a greater focus on the purpose of the study. Midwives accessed a different set of vignettes with open-ended questions and prompts relevant to their role. Participants were presented with four scenarios that represented their experiences of service provision, perceptions of the influence of the organisational structures, processes, and people at the consultation for DS screening on decision-making processes.

Participants without personal access to the Internet were introduced to free Internet services available in public libraries. NHS ethical approval was obtained before the commencement of data collection (reference 11/YH/045). All data for analysis were extracted into a secure web-based database with password access.

### 2.2. Data Analysis

The data were printed directly from the web-based database and manually coded using highlighters. A combination of content and principles of grounded theory analyses [56] was used to analyse the data. The data were initially read in full and deductively coded into broad categories based on the topics of the scenarios [57]. The unit of analysis used was line-by-line coding with single words, phrases, and sentences closely examined to give them labels known as meaning units [58, 59]. Next, meaning units were inductively sorted into categories. The meaning units were then grouped into subcategories. These were refined until no new categories emerged from the data and satisfactorily agreed on by the authors. Using the “analytic power” of categories, the patterns and the relationships between categories were explored through constant comparison analysis approach to ensure interpretative rigour [60, 61]. Finally, overarching concepts, reflecting important organisational issues (i.e., power, routinisation, and tensions), were developed from both maternity services.

## 3. Results

Thirty-four service providers, community midwives (MW), 35 pregnant women (W), and 15 partners (P) completed the online interviews. Some women did not participate in the interviews whilst their partners did and vice versa. The reasons for nonparticipation are unknown. Participants' sociodemographic characteristics are summarised in Table 1. The quotations have identifying numbers; that is, D represents district maternity service, C stands for city maternity service, and they are cited verbatim.

When asked to detail their experiences and perception of the influence of service organisation and delivery on decision-making processes, participants described complex, multifaceted, and interdependent issues such as information overload, asymmetric power relations, the influence of the ambience of the environment, workload pressure on providers, and providers' pressures on women to screen. The concepts of organisational constraints, power, routinisation, and tensions were most commonly identified from their descriptions. It was clear that service users and providers felt that these organisational issues which did not occur in isolation influenced their perspectives and participation.

After all that info piled upon a newly pregnant woman in 50 minutes, when you get to the end and asking about Down's screening, of course the answer will be an uninformed yes as she will be tired/hot/stuffy room and brain-dead. Clinical setting = tests = blood = scans = being a good patient = saying yes to everything. Hard to say no, hard to ask questions. Uniforms are a barrier in my opinion. They say ‘nurse who does some tests on me'. Uniform says I would like a barrier between us. Uniforms = ‘I know best' this environment is scary and I think people will take any tests that may even be adhered to. D1 (MW)

It is a clinical setting and people may feel that they are pressured into screening as this is for the best. With the push for women to accept Down's screening they may feel like they have no choice but to do so. C1 (W)

**Figure 2:**
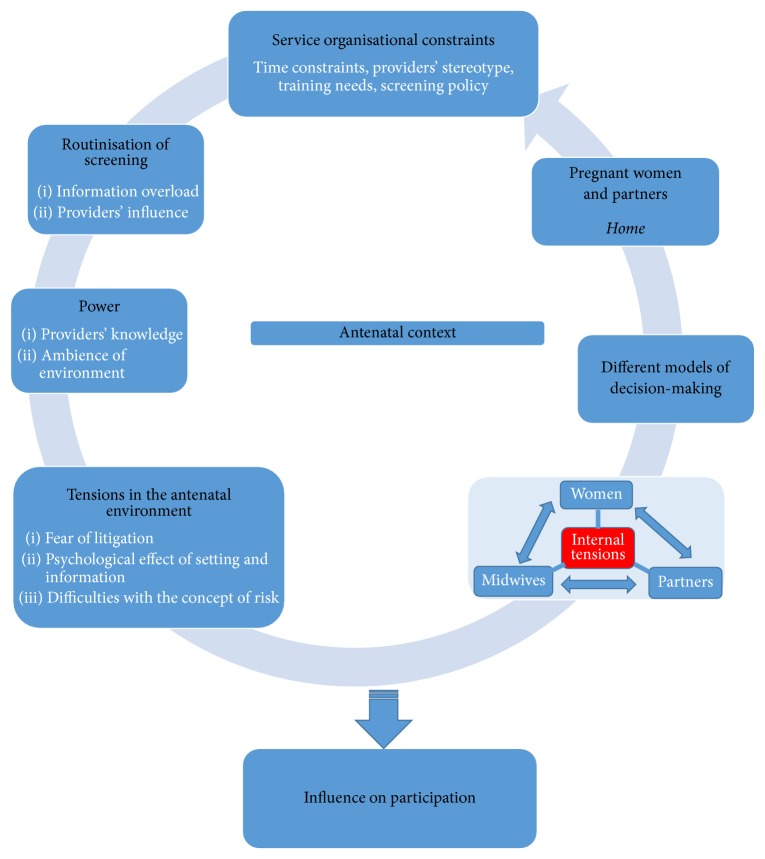
A conceptual framework showing participants' perceptions of the influence of service delivery on participation in antenatal Down's syndrome screening.

Focusing on these multifaceted and interdependent organisational issues was a useful way to capture providers and users' experiences and influences on participation in DS screening. It also helped develop a conceptual framework (Figure 2) that may bridge the gap between research findings and policy development.

### 3.1. Organisational Constraints

It was evident that the range of experiences and interpretations that service providers and users had of the organisation and delivery of screening influenced their participation in the programme. They described organisational constraints such as time pressure, provider's beliefs, and unmet training needs.

#### 3.1.1. Time Constraints

Most providers in both maternity services described how they struggled to inform women about screening due to time pressure, compounded by volume and type of caseload encountered. Some offered the same minimum information to all users.

Time restraints are always a problem. I feel there is not enough time to give all the information necessary to help them make an informed choice at the first appt where they have to decide whether they want Down's screening. D2 (MW)

I keep the information basic and give them the leaflet…I give the same information to all women. C1 (MW)

The following user's report supports the view that some women participate in screening without verbal information, went home, returned when pregnant again, and participate without verbal information. It denotes pressure on users to participate in screening with or without understanding of the information about screening. In addition, time constraints may have pushed providers to ignore the values, beliefs, and varying information needs of users.

I was told about the need to have the baby screened for Down's syndrome and basically given a leaflet and booklet about the condition. In all my pregnancies, blood samples were taken for screening without detailed verbal information from the midwife. D1 (W)

#### 3.1.2. Providers' Beliefs

Many providers in district maternity service compared with city maternity services noted that women, particularly those from ethnic minority groups, declined screening for cultural reasons. These collective implicit beliefs may have affected the way information about screening was presented to users.

Many of the women I look after are migrants, who don't speak English or struggle with complexities of the English language…many women decline the test because of cultural preferences. D3 (MW)

However, the comments of pregnant women from ethnic minority groups illustrate that providers' beliefs may be stereotypical.

In my culture children with Down's syndrome are stigmatised and generally looked down upon and sometimes even killed…This made me to consider having the screening as I will be able to make a decision on whether to or not to go ahead with the pregnancy. D1 (W)

#### 3.1.3. Unmet Training Needs

Some providers described lack of skills to check users' understanding of information at booking as a constraint on their ability to effectively inform about screening. Apparently, learning through experience may be confusing and conflicting to providers as they are not taught or trained to check users' understanding of information.

Midwives also need the skills to get women to repeat back information in order to check understanding, this is not taught. D4 (MW)

### 3.2. The Influence of Routinisation

This was related but not limited to the combination of information overload and providers' influence.

#### 3.2.1. Information Overload

The majority of users in city maternity service described the information given at booking as overwhelming. Similarly, providers also felt that the amount of information given at booking affected users' perception of the DS screening information.

Felt a little overwhelmed by all the advice I was given. C1 (P)

There is a lot of information to take in…. C1 (W)

The amount of information given in one allocated appointment I feel trivialises the importance and significance of the screening test and relegates it to routine and therefore can be perceived as not needing special thought or consideration…. C2 (MW)

#### 3.2.2. Providers' Influence

Users pointed out that providers' implicit or explicit manner of presenting information about screening informed their preferences. They explained that the offer was often not linked to the implications of screening; that is, it may involve termination of affected pregnancy. In many instances, screening was offered as a routine test. Providers also pushed boundaries by encouraging or discouraging participation.

I believe the reliability of the nuchal test compared with the triple test encourages women to have the test. The unreliability of the triple test made it more likely for the midwife to impose her own views on the test. D5 (MW)

Yes, midwife explained that it was commonly done, a routine test. C4 (W)

### 3.3. Ambience of the Clinical Environment (Power)

Some users detailed how the ambience of the clinical setting and trust in their providers' expertise influenced their decision-making processes. They felt that the seating arrangement, warm relaxed atmosphere in the consulting rooms, and the superior knowledge of providers were influential to their participation in screening. It was apparent that they found the process disempowering while others chose to be passive.

It was warm and relaxed in the room we had, it gave me a sense of trust in the person we were talking to; if I had been in a dingy room with someone who hadn't a clue what they were telling us about we would have been inclined to move to another care provider and would probably have looked further into this ourselves. C7(W)

…*when you are being addressed by someone in uniform in an environment where you perhaps feel that power is taken away from you, you're more likely to feel that you've been told to do something rather than discussing something for you to make your own mind up on. C2 (W)*

This may be associated with providers' ability to interpret complex technical and probabilistic risk information that was often not fully understood by service users, indicating power differentials in knowledge, consequently reinforcing the routinisation of screening.

No explanation just briefly mentioned it; still don't know what the blood tests I have had done are for. C3 (W)

### 3.4. The Creation of Tensions

Providers and users' differing perceptions of emotional effects arising from information, beliefs, expectations, and dissonance between stated policy and its implementation created tensions.

#### 3.4.1. Tension amongst Users

*Emotional Effect of Information*. In response to the interview questions about how users respond when provided with information about DS screening, providers pointed out that some women looked terrified when informed about the possibility of their babies having the condition and overwhelmed when asked for a decision about screening. In addition, most users reported feeling scared, terrified, and anxious by the information. Hence, information about DS screening created tensions in users.

The women look a little shocked to be given this information. D18 (MW)

Scared, on top of all the information you are given at your first midwife appointment it can be a lot to take in…. C6 (W)

Further, partners' comments suggest the term Down's syndrome generated tensions.

I think the word Down's syndrome test itself brings a negative ring…. D1 (P)

*Partners' Influence*. Some pregnant women believed their partners' insistence on screening had precedence over their preferences not to undergo screening reflecting pressure to agree to screen.

I had the screening done since it was my partner's wish…. C7 (W)

#### 3.4.2. Tensions amongst Providers

There were nuances in the operationalisation of screening guidelines in the antenatal context. For example, the data revealed discordance between the programme's goal of nondirective informed choice and the actions of recommending and booking screening appointments for women. The policy was viewed as a “rule” and contradictions in its implementation appeared to create tension amongst providers.

Due to the implementation process a decision is required immediately/at referral therefore if there is some indecision it is more common to recommend screening and decline later…. D6 (MW)

Tensions also occurred when interpreters and users lacked understanding of the concept of risks. The interplay between organisational constraints such as time pressure and providers' lack of skills to check users' understanding reinforced tension.

*Interpreters' Impact. *Some providers in district maternity service revealed that interpreters lacked understanding or grappled with the complexities of the concept of Down's syndrome screening, in addition to slowing down the process of informing users.

Interpreters slow the process down. ID204T (MW)

Also difficult when there are language barriers because even with interpreters who themselves are not sure what Down's syndrome is. D9 (MW)

*User's Lack of Understanding. *Providers described their concerns that users often did not understand the information about screening which meant their consent to screen may not be informed.

I think screening is an important subject but I am not always sure if all the women totally understand what they are saying yes or no to. C3 (MW)

#### 3.4.3. Tensions between Providers and Users

Perceived inadequate and rushed information-giving and perceived providers' expectations created tensions between service providers and users. Providers and users' reports of their experiences indicate different agendas, providers and partners' pressures that expose women to interdependent organisational pressure.

*Information Is Rushed*. Providers' acknowledged that rushing through information-giving about screening influenced women to accept screening. Some service users felt their freedom to freely decide was threatened by the way information about screening was delivered and the implicit providers' expectation.

Due to time constraints I feel I can sometimes speed through the delivery of the information and then clients just agree to participate. C4 (MW)

Felt that explanation was a bit rushed as if I was expected to partake in the test. C10 (W)

Time pressures means you are sometimes having to rush…. D8 (MW)

In addition, the comments of users indicate tensions exist between their desires to avoid harm to their babies and the focus of providers to offer screening or detect abnormality.

No did not want a test which would mean my wife would have to consider a further test to see if baby was Down's and could kill the baby by doing so. Prefer not to know. C2 (P)

#### 3.4.4. Tensions in the Antenatal Context

Providers' description of their fear of litigation and the measures undertaken to address such risks was an indication of the tensions experienced in the context of screening. They also believed the antenatal environment where screening was offered provoked anxiety, helplessness, and coercion on users' decision-making processes

*Fear of Litigation*. Some providers adopted a defensive approach as women who declined information about Down's syndrome screening at booking were requested to document it.

If they do not wish me to impart the information I get them to sign they have declined in case later they said I did not offer them the information! D9 (MW)

*Psychological Impact of Environment*. Several providers explained that some users' became “medicalised” or displayed “white coat syndrome” in the antenatal context. The terms describe a reluctance to actively ask questions of providers. It describes how the settings created anxieties or tensions in users. This is similar to “white coat hypertension,” a situation where patients experience transient elevation in blood pressure due to the presence of a physician.

Although we try to make the environment conducive I am sure many clients get ‘white coat syndrome' and feel obliged to agree to any screening. C5 (MW)

The pressures experienced by providers and users in the antenatal context drive the interdependency of these organisational issues on decision-making processes.

…discussing and consenting a woman to Down's syndrome screening is a lot to do in the first booking appointment, both in terms of time and pressuring the woman to make a decision. D10 (MW)

I feel clients just go along with everything and may feel pressured to accept all tests as routine. C5 MW

### 3.5. Decision-Making Models

The comments of some users indicated they gave consent to the preferences of providers. Some providers supported users in the decision-making processes by exchanging information based on assumed beliefs, values, and circumstances of the users. In other cases, providers offered information about DS screening and stayed out of the decision-making processes. These denote a combination of paternalistic, informed, and shared decision-making processes in a programme that has a policy of autonomous informed choice.

I was told to have it by midwife…. C8 (W)

Yes, I feel the midwife was very professional and comforting, I didn't feel as though I couldn't approach her and ask, I feel I was able to freely express any concern I had and she answered with information helping me understand more. D2 (W)

I wasn't influenced by anyone or anything, only our choice as a couple. C9 (W)

## 4. Discussion

This study aimed to explore the influence of service organisation and delivery on participation in Down's syndrome screening. The study developed a conceptual framework from five emergent main concepts based on providers and users' perspectives on organisational issues encountered in the antenatal context: constraints, power, routinisation, tensions, and different decision-making models (Figure 2).

The developed framework provides key points at which the structure and process of service delivery shape participation. For example, providers in district maternity service believed that users from ethnic minority backgrounds decline screening for cultural reasons. These collective, implicit providers' stereotypical beliefs and time constraints suggest informal organisational constraints that shape providers' practices and may affect the way DS screening was presented to users. However, these assumptions are challenged by the literature on DS testing [62, 63] and the wider literature [64]. Other influences include how screening was presented as a routine test, sometimes because of limited time, rather than an optional test, which indicate routinisation of screening. Other authors have reported similar findings [20, 65, 66]. In contrast, some users detailed how the space and layout of the consulting room and trust in their providers' expertise influenced their decision-making processes. These reports generated the concept of power amongst providers in the antenatal context. The finding is also consistent with the wider literature [67]. However, previous research suggests [68, 69] that women's account about the routine nature of screening and providers' expert authority in the antenatal context indicates that the complex decision-making processes involved in screening were circumvented, because informed consent could not have been obtained from pregnant women without a sense of choice. Furthermore, service users in both study maternity services reported being explicitly directed by providers to participate in Down's syndrome screening. Obviously, this indicates organisational pressure to agree to screening, which may reinforce routinisation of screening, the expert status of service providers, and generate misunderstandings and tensions in the antenatal context.

The interview data suggest that information about screening aroused strong emotional reactions from users. These were threatening thoughts about having a baby with Down's syndrome. Therefore, foetal screening for Down's syndrome generated tensions in users. This suggests the offer of screening is associated with some difficulties, including complex information about risk and unsure anticipation, which may lead to ethical dilemmas and psychological stress [70, 71]. Farrell et al. [72] suggest that anxiety generated in antenatal settings may result in women becoming less thoughtful or having impaired ability to acquire, recall, and synthesize information about screening. Women's acceptance or rejection of screening would then be based on decisions made from the context rather than from the content of the information given as noted in existing work [73, 74]. Additionally, the finding that providers in district maternity services recommended screening to undecided users was unexpected due to the nondirective informed choice screening policy. This in combination with the fear of litigation, differing perceptions about users desire to avoid harm to their babies and the focus of providers to offer screening, and difficulties with the concept of risk created tensions amongst and between providers and users. Tensions have been mentioned in prior research on DS screening which called for improvement in the midwife-woman communication [10, 16, 24, 75, 76] and in the medical education literature [77, 78].

Consequently, these organisational constraints and tensions could account for the different decision-making models seen in the antenatal screening context. Some women were given written information but no verbal information about screening and were requested to make a decision indicating an “informed choice” model that is not in its purest form. Shared decision-making was demonstrated when midwives engage in a dialogue whilst taking assumed beliefs, values, and life circumstances of the women into consideration in the decision-making process. Shared decision-making recognises the autonomy of the pregnant woman and that the final decision lies with the pregnant woman [79, 80]. However, paternalistic decision-making was revealed in both study maternity services when midwives directed pregnant women to have the screening test done, without providing the opportunity for them to decide on their own. This indicated that women were passive in decision-making with her involvement limited to that of consent to the preferences of the midwife. The paternalistic model is no longer adopted in healthcare settings, owing to the fact that service users can become autonomous and make informed healthcare decisions when adequately supported [81, 82]. Regarding the home, after deciding on whether or not to screen in the antenatal context, pregnant women and their partners return home. However, new and old users encounter the same organisational issues when they return to the antenatal context for their booking appointments.

Ultimately, these organisational issues may account for the variation in uptake rates of screening seen this current study setting (22.7% and 73.9%). Nonetheless, the qualitative study design and the small sample size make this suggestion difficult to support. Variation in uptake rates of Down's syndrome screening is not important as long as women's decision-making processes are informed. Crucially, this current study found multifaceted organisational influences on participation in screening which require further large national research using multilevel (hierarchical) modelling to verify the developed framework.

### 4.1. Strengths and Limitations of the Study

Adopting a qualitative approach allowed participants to express views that were important to them and the identification and exploration of how different organisational issues in the DS screening contexts interacted and influenced users' decision-making process.

The challenge with using online scenarios is that participants may respond to the questions, based on what they know to be the correct answers and not actually how they behaved in the antenatal context. In addition, selection bias may have been introduced into this study, as a purposive sampling method was employed. Those who participated in the study might have different views from those who did not, as it is possible that dissatisfied providers and users may have completed the online interviews. However, the purpose of this current study was to explore organisational issues affecting participation and ultimately informed decision-making in the antenatal context. Furthermore, there were variations in the responses from participants which may indicate the online methods minimised the bias. The application of the Donabedian framework was useful to shape the study, but not helpful in providing an understanding of the study findings. This may be due to the underlying reality of complex decision-making processes that users experience, which cannot be elucidated if the Donabedian model is viewed as linear rather than cyclical.

These findings represent the views of a small number of service providers and users in England. This weakness was counterbalanced by inviting all service providers and users in the two large maternity services who met the inclusion criteria to participate in the study, which ensured diversity of participants and responses (i.e., age, length of time working, and ethnicity). However, this may have introduced self-selection bias, but the purpose of this qualitative descriptive study was not to generalise findings to the target population, but of theoretical generalisation (transferability). The findings may be transferable to similar contexts but cannot provide any insight into the prevalence of the organisational issues in the DSS context. However, the low response rates of participants in this study may be because the researcher did not have direct access to the midwives. Additionally, many of the participant information sheets given out to women and their partners were obtained after the brief introduction of the research by the sonographers without the researcher's input. The users claimed to either be in a hurry or have another appointment.

To boost the online response rates in future studies, researchers could explore having direct access to all participants where possible. More NHS Trusts from the low and high uptake range could be selected and included in the study. Internet enabled laptops or tablets could also be made available to participants who agreed to take part in the research and are willing to complete the interviews at the point of recruitment. Another approach is to provide incentives for each participant group. For example, participants could be entered in a draw for a general prize such as a gift voucher for an iPad or tablet.

### 4.2. Implications for Policy and Practice

The evidence of multifaceted and interdependent organisational issues, clear relationships with outcomes of users' decision-making process and description of pressures that push users into these constraints, have implications for policy and practice. It suggests that implementing the screening policy/guidelines in the antenatal context proved to be challenging. To improve service provision and overall psychological and wellbeing outcomes of providers and users, there must be a reduction in the dissonance between stated policy and its implementation in the antenatal context. Consistency in the implementation of the screening guidelines/policy would be beneficial. This may enhance equitable provision and context for decision-making. Furthermore, adopting an approach where users are supported to consider the information to achieve informed preferences would meet these aims. The current study highlights the need for prior information preferably before the booking appointment to all women and their partners. When the information is provided again at booking, it may aid comprehension and active engagement in the decision-making processes. Information given in schools, healthcare settings, and wider social networks has been advocated by Lewando-Hundt et al. [83].

Ideally, the booking appointment should be divided into two separate visits. This has been suggested in the NICE guidelines [2]. Adopting two separate visits may reduce tensions in the antenatal context and pressure on women to decide whether to screen. Information about screening may be introduced in the first booking visit. Women and their partners are then given written information and directed to an online decision aid. This will enable women and their partners to discuss and assimilate information about screening. A shorter decision aid could then be used at the second visit to facilitate the informed decision-making process.

Additionally, providers require training on methods to check users' understanding to help ensure an understanding of key information about DS screening. The “teach-back” method [84] provides a way through which providers can check that they have clearly communicated information about screening to users including those with limited health literacy. This research offers further support to the government's proposal to promote high quality care, drive efficiency, and support patients choices in the NHS [8, 11].

## 5. Conclusions

This is the first study that has developed a framework to comprehensively describe the pathways of the influence of service organisation and delivery on users' decision-making processes in the context of DS screening. The framework also provides new insights for intervention at different levels of the screening program to improve service delivery, but more research is required to verify the framework. Nevertheless, the organisational issues identified from the data suggest an urgent need for consistency in the implementation of the screening guidelines/policy in the antenatal context. This may enhance equitable provision and context for decision-making. However, a move to an alternative social model of care that engages providers and users in a process that supports women's decision-making to achieve informed consent may be more appropriate to foster personalised care.

## Figures and Tables

**Table 1:** Demographic characteristics of online interview participants regarding the influence of service delivery on participation in screening.

	District maternity service, number (percent)	City maternity service, number (percent)
Community midwives	15	19
*Age range in years *		
21–34	3 (20%)	6 (32%)
35–54	12 (80%)	13 (68%)
*Work experience, range in years*		
0–20	3 (20%)	10 (57%)
21–40	12 (80%)	9 (43%)
*Ethnicity*		
White British	15 (100%)	17 (89%)
Any other ethnic group	—	2 (11%)
Pregnant women	16	19
*Age range in years*		
16–34	2 (12.5%)	14 (74%)
35–54	14 (87.5%)	5 (26%)
*Education *		
No formal qualifications	—	2 (10.5%)
GCSE	2 (12.5%)	7 (37%)
Diploma	5 (31.25%)	5 (26%)
First degree	5 (31.25%)	3 (16%)
Postgraduate	4 (25%)	2 (10.5%)
*Ethnicity*		
White British	6 (37.5%)	16 (84%)
Any other ethnic group	10 (62.5%)	3 (16%)
Partners	7	8
*Age range in years*		
16–34	1 (14.3%)	6 (75%)
35–54	6 (85.7%)	2 (25%)
*Education*		
GCSE	2 (28.6%)	2 (25%)
Diploma	—	4 (50%)
First degree	—	1 (12.5%)
Postgraduate	5 (71.4%)	1 (12.5%)
*Ethnicity*		
White British	1 (14.3%)	7 (87.5%)
Any other ethnic group	6 (85.7%)	1 (12.5%)
